# The Effect of Feed Form on Diet Digestibility and Cecal Parameters in Rabbits

**DOI:** 10.3390/ani7120095

**Published:** 2017-12-07

**Authors:** Isabella Corsato Alvarenga, Charles Gregory Aldrich, Micah Kohles

**Affiliations:** 1 Department of Grain Science and Industry, Kansas State University, Manhattan, KS 66506, USA; isacorsato@ksu.edu; 2 Department of Veterinary Medicine and Biomedical Sciences, University of Nebraska, Lincoln, NE 68510, USA; micah.kohles@neb.vet; 3 Flatwater Veterinary Group, Lincoln, NE 68510, USA

**Keywords:** rabbits, digestibility, acid insoluble ash, diet form, pelleted, extruded, muesli

## Abstract

**Simple Summary:**

In addition to hay or forage in the diet pet rabbits are commonly fed a supplementary food as a muesli (granular mix), pellets, or extruded croquettes. This study aimed to determine if form of this supplementary diet (pelleted vs. extruded) or composition (muesli) had an effect on the diets total tract digestibility and cecal fermentation patterns. Rabbits had slightly higher intake when fed extruded and pelleted diets compared to muesli. Digestibility results were inconsistent between estimation methods. The extruded diet was more digestible than pelleted according to the total collection digestibility method, but according to internal marker acid insoluble ash the pelleted diet was the most digestible. Both the extruded and pelleted diet had similar fermentation patterns, with lower cecal pH and greater proportions of butyrate. Our findings suggest that diet composition, rather than form, may have a greater impact on nutrient utilization by rabbits.

**Abstract:**

Fifteen New Zealand rabbits were randomly assigned to one of 3 dietary treatment groups of 5 animals each and fed pelleted, extruded, or muesli diets in a completely randomized design experiment. Rabbits were placed in individual cages with *ad libitum* access to water and food for 45 days acclimation followed by 30 days experimental period. Feed intake of rabbits fed pelleted and extruded diets was greater (*p* < 0.05) than rabbits fed the muesli diet (125.6 and 130.4 vs. 91.9 g/d), but weight change and feed efficiency were not affected by treatment. Diet digestibility among the treatments was inconsistent when comparing results obtained from total fecal collection and AIA (please define) as an internal marker. Rabbits fed extruded and pelleted diets had lower (*p* < 0.05) cecal pH (6.42 and 6.38 vs. 7.02, respectively), and higher (*p* < 0.05) production of SCFA (18.5 and 19.0 vs. 11.7 mM, respectively) than those fed muesli. The fermentation products from rabbits fed pelleted and extruded diets had a greater proportion of butyrate and less propionate than rabbits fed muesli. The results of this study indicate that the basal dietary composition had a greater impact on diet utilization and cecal fermentation than food form.

## 1. Introduction

Most domestic pet rabbits are fed a staple diet of dry forages such as long-stem grass hays (i.e., *Phleum pretense* (Timothy) or *Dactylis* (Orchard) grass hay) or loosely baled alfalfa (*Medicago sativa*) and a supplement based on hay and grain fortified with vitamins and minerals [1]. These supplement feeds can be found in various forms. Some are loose meal, granular rations, or muesli consistency, many are pelleted, and a few products utilize extrusion processing to form a croquette or kibble. Claims regarding the value of one form over another have been made regarding digestibility, feed utilization, dental malocclusion, animal health, and overall nutrition. However, very little data is available to substantiate these positions. Pelleted feeds are the most common supplemental form in the United States. This process effectively agglomerates vitamins and minerals along with fibrous byproduct feeds and some starchy grains into a single feedstuff. It provides convenience; helps avoid ingredient separation and aids uniformity of nutrient delivery to the animal.

To avoid digestive upset it is recognized that rabbits require complete digestion of starches before the cecum and fiber for stable cecal fermentation [2]. Extrusion of rabbit diets is less common than pelleting; however, extrusion has been reported to decrease the amount of starch reaching the ileum [3]. Due to its added cost and limited benefit to the performance of production rabbits [4] pelleting has remained the predominant product form over extrusion. There is need for more studies regarding how food form may impact nutrient utilization and gut health in the pet rabbit. Therefore, the objectives of the study were to determine the effect of food composition or form on diet utilization and cecal fermentation in adult rabbits.

## 2. Materials and Methods

### 2.1. Animals and Diet

Fifteen New Zealand white rabbits, males and females approximately 6–7 months in age were randomly separated into three groups of five animals each. There were at least two animals of each gender in each group. The three treatments consisted of rabbits fed pelleted feed, extruded croquettes, or muesli mixed diet (Table 1). Rabbits were fed an amount of food intended to maintain their body weight, orts were weighed daily and recorded, and no hay or other forage was offered. The experimental diets were commercial foods. The pelleted and extruded diets were of the same composition and consisted predominately of timothy grass meal, soybean hulls, soybean meal, cane molasses, wheat middlings plus functional and nutritional additives. The muesli experimental diets consisted of a much broader range of ingredients including timothy hay, alfalfa hay, oats, soybean meal, oat hulls, ground oats, wheat middlings, ground wheat, sunflower seed, wheat, barley, oat groats, shelled peanuts, ground corn, ground flaxseed, dehydrated carrots, raisins, dried bananas, dried papaya, safflower seed, ground rice, dried pineapple, dehydrated sweet potatoes, pumpkin seed, dried cane molasses, dehydrated apples with functional and nutritional additives. No supplemental hay or forage was fed because it was a short-term study and anything rabbits ingested besides the diets could confound digestibility results.

Water was available *ad libitum* throughout the study. No measures were taken to prevent cecotrophy. Rabbits were acclimated to the new diets for 42 days followed by 30 days sample and data collection. Each rabbit was housed individually in suspended wire cages with solid floor resting pad and nest box in a ventilated metal building. The study was conducted at a USDA inspected Rabbitry in Alabama (USA) consistent with meat-rabbit production and no institutional animal care committee oversight was required. Body weights and feed intake were measured weekly throughout the study. Representative samples of feed and feces were collected. Feces were dried in an oven at 55 °C until dry to the touch, then ground and stored in plastic bags at 4 °C until analyses were performed. At the conclusion of the experiment rabbits were humanely euthanized and further evaluated post mortem for cecal fermentation parameters.

### 2.2. Sample Analysis

Feed and fecal samples were dried in a laboratory oven for 24 h at 60 °C to determine moisture (AOAC 930.15). Dried samples were ground using a laboratory grinder (Retsch, Newtown, PA, USA) with a 0.5 mm screen size. Crude protein was determined by combustion analysis (LECO; St. Joseph, MI, USA). Crude Fiber was determined by the Wende method [5] and neutral detergent, acid detergent fiber (NDA, ADF) and acid detergent lignin (ADL) were determined according to Van Soest et al. (1991) [6] after pre-treatment with termamyl (amylase). Starch was analyzed by a glucoamylase digestion to glucose then quantified by YSI (Yellow Springs, OH, USA; AACCI, no. 76–13) spectrophotometer. Gross energy was determined by Parr adiabatic bomb (Moline, IL, USA).

### 2.3. Feed Efficiency and Fecal Output

Feed efficiency was determined by dividing the total weight change in the 30-day collection period (final weight-initial weight) by the total feed intake during 30 days (Table 2).

Fecal output of nutrients (in grams; Table 3) were calculated by multiplying total fecal output on a DM basis by the concentration of each nutrient in either food or feces. Dry matter is used as an example below:

DM fecal output=total fecal output on DM basis × DM % in feces

### 2.4. Total Fecal Collection Apparent Total Tract Digestibility

Feces were collected the last 96 h prior to the conclusion of the study, and feed intake was recorded over this same period. Samples of feces were collected into whirl-pak bags (Nasco Inc., Fort Atkinson, WI, USA) and stored frozen (−10 °C) until processing and nutrient analysis was possible. Total fecal collection (TFC) digestibility (using DM as an example) was calculated using Equation (1):

(1) $$\mathrm{Apparent}\;\mathrm{Total}\;\mathrm{Tract}\;\mathrm{Digestibility}=\frac{\left(\mathrm{intake}\;\mathrm{DM}-\mathrm{feces}\;\mathrm{DM}\right)\times 100}{\left(\mathrm{Intake}\;\mathrm{DM}\right)}$$

The digestibility of organic matter, energy, crude protein, crude fat, NFE, starch, crude fiber, ADF, NDF, lignin and ash were calculated in the same manner (Table 4).

### 2.5. Acid Insoluble Ash Fecal Output and Digestibility

The method for determination of AIA was based on a procedure developed by Van Keulen and Young (1977) [7]. Wherein, 4 g of fecal or 10 g of food samples were ashed, then boiled in 2 N HCl. This was filtered with ashless filter paper, and ashed again to burn the paper and obtain the weight of AIA. The AIA digestibility was calculated using the following equation:

(2) $$\mathrm{AIA}\;\left(\%\right)=\frac{\left(1-\left(\%\;\mathrm{of}\;\mathrm{nutrient}\;\mathrm{in}\;\mathrm{feces}\times \%\mathrm{AIA}\;\mathrm{in}\;\mathrm{feed}\right)\right)\times 100}{\left(\%\;\mathrm{of}\;\mathrm{nutrient}\;\mathrm{in}\;\mathrm{feed}\times \%\mathrm{AIA}\;\mathrm{in}\;\mathrm{feces}\right)}$$

### 2.6. Cecal Analysis

Samples of cecal contents were collected from each rabbit (*n* = 15) into whirl-pak bags (Nasco Inc., Fort Atkinson, WI, USA) immediately after the animals were euthanized. Consistent with the procedures outlined by Belenguer et al. (2005) [8], the cecal contents were immediately measured for pH with a glass electrode pH-meter. From these samples, two 1 g aliquots of cecal contents were acidified with 0.2 mol/L HCl and 0.15 mol/L H_3_PO_4_ into 50 mL disposable centrifuge vials and stored at −20 °C pending determination of ammonia and short chain fatty acids (SCFA), respectively. Samples for VFA and NH_3_ analysis were mixed 1:1 with a 25% solution of meta-phosphoric acid and frozen, then separated into supernatant and analyzed according to the procedures of Mullins et al. (2007) [9]. Cecal VFA concentrations were determined using the procedure of Jouany (1982) [10] for gas-liquid chromatography, and ammonia concentration with the procedure by Chaney and Marbach (1962) [11].

### 2.7. Statistical Analysis

The study was conducted as a completely randomized design (CRD) with rabbits randomized to treatment. Rabbit served as experimental unit with five animals per dietary treatment. Means were separated by Analysis of Variance (ANOVA) using the general linear model (GLM) procedure from the statistical analysis software (SAS v 9.4, SAS Institute Inc., Cary, NC, USA). Means were considered different at a *p* < 0.05.

## 3. Results and Discussion

### 3.1. Diet Composition

The pelleted and extruded diets were of similar ingredient composition and differed from the muesli diet (Table 1). This followed in their nutritional composition as well. The products were commercial dry foods and DM was similar among dietary treatments, averaging 94.7%. The Muesli diet was higher in Gross energy (4146 kcal/kg) than either extruded or pelleted diets (4002 and 3981 kcal/kg). This was driven in large part by the muesli diets greater concentration of crude protein, crude fat, and starch relative to the pelleted or extruded diets (17.1%, 5.93%, and 24.55% versus an average 14.2%, 2.62%, and 1.04%, respectively). Calculated starch level as NFE was ranked similarly among the diets, but did not exhibit as wide a spread between the muesli versus the pelleted and extruded diets (51.8% vs. average 42.8%, respectively). These differences may be a function of nonstructural carbohydrates (soluble sugars, soluble fiber, and oligosaccharides) that the crude fiber component of the NFE calculation fails to capture. Further, the ADF and NDF of the pelleted and extruded diet were a much larger component of the diet than measured in the muesli treatment. This points to these treatments greater proportion of Timothy hay and less grains and grain products noted in the ingredient list when compared to the muesli diet. Finally, the ash content for the muesli dietary treatment was lower than the pelleted or extruded diets (5.79 vs. average 8.30%, respectively), but the AIA did not correspond directly to treatment with an average of 1.47% among the diets.

### 3.2. Intake, Weight Gain and Fecal Ouput

This study utilized commercial meat type rabbits as a model for companion rabbits. In commercial rabbit production the goal is to raise 1.8 to 2 kg fryers in 8 weeks [12] and the diets fed are intended to support rapid weight gain [13]. However, chronic overfeeding in pet rabbits may predispose the animal to obesity and gastrointestinal disturbances [14]. In this study, food intake was fixed to a level that was intended to maintain body weight and rabbits were fed the experimental diets without supplemental forage. The feed intake of rabbits fed the muesli diet was lower (*p* < 0.05) than those fed the pelleted and extruded diets (91.9 vs. 125.6 and 130.4 g/day, respectively) based on initial estimations (Table 2). For instance, rabbits had a similar protein intake: average (15.7, 17.7 and 18.5 g/day, for muesli, pelleted, and extruded diets, respectively). However, actual energy intake deviated from initial estimated iso-energetic values (e.g., 381, vs. 500, 522 kcal/day for muesli vs. pelleted and extruded). The resulting body weight gain, and feed efficiency were unaffected by treatment most probably a function of the large variation between rabbits body weights toward the end of the study.

By the total fecal collection (TFC) method for estimating fecal output, rabbits fed the pelleted diet produced more DM excreta than those fed the extruded diet, and in turn each defecated more than those fed the muesli diet (58.6 > 43.6 > 26.7 g/d; *p* < 0.05; Table 4). Fecal output of organic matter, energy, crude protein, crude fat, and NFE, crude fiber, ADF, NDF, ash followed the same order as DM fecal output (*p* < 0.05). The amount of starch excreted per day was very low and did not differ among treatments (average 0.051 g/d). Lignin output was greater for rabbits fed pelleted (4.89 g/d) than muesli or extruded treatments (average 3.20 g/d). The AIA fecal output was greater (*p* < 0.05) for rabbits fed pelleted (2.80 g/d) than those fed muesli or extruded diets (average 1.46 g/d).

A common premise for use of markers is that they are non-digestible and non-absorbable in the gut [15]. Therefore, internal markers like AIA should have similar inputs and outputs by rabbits fed each diet. In this study AIA food intake and fecal output of muesli and extruded dietary treatments were similar (1.27 g/d and 1.36 g/d for muesli and 1.80 g/d and 1.57 g/d for extruded; input and output, respectively). However, AIA intake versus output of rabbits fed the pelleted diet had a difference of almost 35%: 2.08 g/d versus 2.80 g/d, respectively. This means that during TFC rabbits might have ingested more food or excreted less feces than what was collected, and this may have been masked by the ceacotrophic behavior of this species. This illustrates a situation where TFC might not be the ideal method to calculate apparent total tract digestibility.

### 3.3. Total Collection Digestibility

Diametric to TFC results, apparent total tract digestiblity of most nutrients was greater for rabbits fed the muesli and extuded diet than those fed the pelleted diet (Table 4). For example, the dry matter (DM) and organic matter (OM) digestibilities were higher (*p* < 0.05) for rabbits fed the muesli and extruded dietary treatments than those fed pelleted diets (70.9% and 66.3% vs. 53.2% for DM and 72.4% and 67.0% vs. 54.4% for OM, respectively). Within this same range, Nieves et al. (2008) [16] using TFC methods also reported average apparent DM and OM digestibility for rabbits fed pelleted diets of 57.6% and 60.0%, respectively. Crude fat digestibility deviated slightly from this pattern; wherein, rabbits fed the muesli diet had a greater (*p* < 0.05) apparent total tract digestibility than rabbits fed the extruded diet, which in turn was greater (*p* < 0.05) than rabbits fed the pelleted diet (96.3% > 90.7% > 82.9%, respectively). The digestibility of NFE was greater for rabbits fed the pelleted and extruded diets than those fed the muesli (average 54.5% vs. 47.4%, respectively). This differed from the results for starch apparent digestibility which was not affected by treatment (average 97.7%). This nearly complete digestion of starch in contrast to the NFE would suggest that the nonstructural carbohydrate fraction of these diets had a prominent impact on their utilization.

This seems to agree with the apparent total tract neutral detergent fiber (NDF) digestibility which was greater (*p* < 0.05) for rabbits fed the extruded and pelleted dietary treatments than those fed the muesli (59.8% and 46.6% vs. 36.0%, respectively). Lignin digestibility data are difficult to interpret as rabbits fed the pelleted diet apparently excreted more lignin than they consumed, leading to a negative digestibility value. This may be possible with rabbits given their practice of cecotrophy and the timing by which the collection pans were placed and removed.

Total mineral digestibility for rabbits fed the extruded diet was greater (*p* < 0.05) than those fed the muesli and pelleted dietary treatments (60.8% vs. 40.6% vs. 51.2%, respectively).

### 3.4. AIA Estimate of Apparent Total Tract Digestibility

The determination of digestibility by the AIA method was done to provide validation for the results obtained through total fecal collection procedure and to evaluate this method as a future internal marker when collection of all feces is not possible. In general, the digestibility of all nutrients was greater by this method than the results obtained from total fecal collection. Further, the rabbits fed the pelleted diet had a greater apparent total tract digestibility than those fed the muesli or extruded diet. For example, the dry matter and organic matter digestibilities were greatest (*p* < 0.05) for rabbits fed the pelleted, intermediate for muesli, and lowest for extruded dietary treatments (71.5% vs. 67.7% vs. 62.0 % and 72.2% vs. 69.2% vs. 62.6 %, respectively; Table 5). Crude protein digestibility deviated slightly from this theme; wherein rabbits fed the muesli and pelleted diets were greater (*p* < 0.05) than those fed the extruded dietary treatment (average 77.1 vs. 70.8%, respectively). Crude fat digestibility followed level in the diet; such that, rabbits fed the muesli dietary treatment had a greater overall digestibility of fat than those fed the pelleted and extruded diets (95.9% vs. average 89.5%, respectively). The NFE digestibility of rabbits fed muesli and pelleted diets was greater (*p* < 0.05) than that of rabbits fed the extruded diet (76.5% and 76.2% vs. 67.9%, respectively). This compared to near complete digestion of starch with no difference among treatments (average 97.7%). The crude fiber, ADF, and NDF digestibilities declined (*p* < 0.05) in the order of pelleted > extruded > muesli treatments. Lignin digestibility was not affected by dietary treatment (average 9.53%). The ash apparent total tract digestibility was lowest (*p* < 0.05) for rabbits fed the muesli diet.

### 3.5. Correlation between Total Fecal Collection and AIA Apparent Total Tract Digestibility

The TFC method for rabbits has some limitations: there can be cross contamination of feces and urine, it requires significant effort to collect all feces, animals must be confined and this may tend to relax muscle tone, which can in turn slow intestinal transit and lead to an overestimation of nutrient digestibility [16]. This is why markers to estimate fecal output can be important. Using AIA to calculate digestibility is similar to other markers. It requires fecal collection from each animal over a period of time and then quantification of the amount of indicator or marker [17] in the samples. A common marker used to estimate fecal output and thereby calculate digestibility is chromium sesquioxide (Cr_2_O_3_) [18]. However, the results obtained from using Cr_2_O_3_ can be variable [19]. One challenge with Cr_2_O_3_ is that it has to be intentionally introduced into the feed prior to the experiment, whereas AIA is an intrinsic factor in most feedstuffs with a meaningful concentration of naturally occurring ash (minerals). The AIA marker for fecal output and estimation of apparent digestibility has been reported in several studies with rabbits [20,21,22,23].

In the current study, the intent was to corroborate the TFC for determining digestibility with AIA as an alternative marker. It appears there was consistency between the methods for rabbits fed the extruded and muesli diets. However, the results for the rabbits fed the pelleted diet were inconsistent and hard to decipher regarding which method yielded the most accurate results. There is indication that AIA was more accurate. As mentioned before, when looking at AIA fecal output and AIA feed intake, the pelleted treatment had a 35% difference (2.08 vs. 2.80). That means that amount of food and (or) fecal output might have been measured inaccurately. Also, an average 53.2% DM digestibility for the pelleted treatment (Table 4) seems low. A French study conducted in 1980 reported TFC DM digestibility of 61.8% for rabbits fed a pelleted diet [24], a value that is in between these results for TFC and AIA methods.

Some studies have reported that AIA correlates well with TFC. Nieves et al. (2008) [16] concluded that AIA [25] lead to similar results as TFC. Sales and Janssen (2003) [26] reported that this procedure overestimates nutrient digestibility because the HCl soluble minerals can be erroneously accounted as AIA. However, this presumably won’t happen in the procedure of Keulen and Young (1977) [7] where samples are ashed before boiling in acid. This method was also found by the authors [7] to be less time consuming, safer, and less odorous since it uses a lower strength HCl (2N vs. 4N). In a study with horses De Marco et al. (2012) [27] found that DM, OM, gross energy, crude protein and NDF apparent total tract digestibilities of three diets were numerically very similar between TFC and AIA (method by Van Keulen and Young, 1977) [7]. In the present study, the Pearson correlation (*n* = 15) showed that nutrient digestibility was poorly correlated between the methods. Dry matter, energy and crude fat were correlated (*p* < 0.05), but correlations for DM and Energy were low (*r* = −0.582, −0.563, respectively; *r* = 0.810 for crude fat). Further, for reasons stated above the TFC method has some challenges; especially, when conducting studies with rabbits due to their practice of cecotrophy and potential inconsistencies regarding timing and type of fecal excretion (e.g., soft vs. hard feces). Therefore, data obtained from the AIA method would likely be more effective for interpreting the digestibility data from this study.

### 3.6. Diet Processing Effect on Digestibility

The premise of this work was to determine whether processing (pelleting or extrusion) had an effect on diet utilization in the rabbit. In theory processing may increase the digestibility of diets up to a point [28]. Cooking improves starch solubility due to gelatinization or disruption of the otherwise tightly bundled starch crystal [29]. Pelleting is a dry feed process commonly used for production-fed animals—it consists of passing a meal through a conditioner where steam and heat are added, and then the material is pressed through a steel die drilled with hundreds of holes which create pellets as they are expelled [30]. Extrusion is a more complex process that involves thermal and mechanical energies through steam addition and shear forces. Pelleting is not as efficient for cooking as extrusion, and therefore they may affect nutrient digestibility differently. For example, weaning pigs fed an extruded diet had improved average daily gain and feed efficiency when compared to pigs fed a pelleted diet of the same composition [31].

Cooking some starch sources has been shown to improve pre-cecal digestion in the rabbit [3,32]. Especially if starch were to be nearing a threshold for utilization in the rabbits small intestines. This could potentially allow undigested starch to spill into the cecum and disrupt fermentation. Unfortunately, it was not possible to measure pre-cecal starch digestion in this case. By the TFC method (Table 4) dry matter, organic matter, energy, crude protein and crude fat were all digested better by rabbits fed the extruded than the pelleted treatment. The total tract measured starch digestion seems to be near complete and does not differ between the treatments. There was a slight increase in the total tract fiber digestibility (NDF, and ADF) for rabbits fed the extruded diet, with rabbit fed the pelleted treatment being intermediate. Converse to the TFC method, AIA measured higher digestibilities for rabbits fed the pelleted diet than the extruded. Considering the lower variation of the AIA method and the concern that the total collection might not have accounted for all the feces, this study leads us to put greater trust in the AIA data than TFC. Hence, the pelleted form appears to have higher digestibilities.

### 3.7. VFA and Ammonia Concentrations in the Cecum and Cecal PH

Short chain fatty acids (SCFA; or volatile fatty acids as they are often called) are produced by fermentation and can be used as a source of energy in animals, contributing 20–30% of the caloric requirements for herbivorous animals, such as rabbits [33]. Further, the amount of refractory carbohydrates (e.g., fiber and indigestible starch) when fermented in the cecum contribute to microbial protein in the feces [34,35]. This is important for cecotrophic animals such as the rabbit which ingest their own nitrogen rich stool to help meet their protein requirements [10,36]. However, this can lead to an understimation of total tract digestibility.

The cecal pH for rabbits fed the muesli dietary treatment was higher (*p* < 0.05) than those fed either the pelleted or extruded diets (7.02 vs. 6.38 and 6.42, respectively; Table 6). Cecal ammonia concentrations in rabbits fed the muesli dietary treatment were lower (*p* < 0.05) than those fed pelleted diets, and rabbits fed the extruded were intermediate to each extreme (3.6 vs. 4.9 vs. 4.3 mM, respectively). The sum of all individual cecal SCFA’s were greater (*p* < 0.05) for the rabbits fed the pelleted and extruded diets when compared to the rabbits fed the muesli (19.0 and 18.5 vs. 11.7 mM, respectively). This would suggest that there was more active fermentation in the cecum for those rabbits receiving the pelleted and extruded diets. As for the individual SCFA, the acetate molar proportions were not affected by treatment (average 77.8%). The propionate molar proportions were greater (*p* < 0.05) for the rabbits fed the muesli than either the pelleted or extruded diets (10.00% vs. 7.53% and 6.60%, respectively). Cecal butyrate followed a pattern the opposite of propionate; rabbits fed the muesli had a lower (*p* < 0.05) molar proportion of butyrate than rabbits fed the pelleted diet, and those fed the extruded diet were intermediate to each extreme. The cecal branched SCFA’s iosbutyrate and valerate mirror the relationship among treatments seen for propionate; wherein, muesli had a greater (*p* < 0.05) proportion of isobutryate and valerate than rabbits fed either pelleted or extruded dietary treatments (1.03% vs. 0.43% and 0.59% for isobutyrate and 1.03% vs. 0.68% and 0.65% for valerate, respectively). There was no difference among treatments for isovalerate.

If more robust fiber fermentation occurred in the cecum, one would assume that cecal pH would be lower (more acidic), ammonia production would be less, short chain fatty acid production would be higher, and the proportion of those SCFA would favor the butyrate at the expense of propionate [37]. In this experiment the rabbits fed the pelleted and extruded diet displayed a lower pH and higher SCFA production, but not a corresponding reduction in ammonia. Further, the rabbits fed the pelleted and extruded diet had a lower proportion of propionate and greater proportion of butyrate. These results would suggest that the pelleted and extruded diets were more effectively fermented than the muesli diet. Thus, dietary composition rather than the texture or process had a greater impact on the cecal fermentation.

## 4. Conclusions

Intake of the muesli diet was lower than that of pelleted and extruded rabbit diets, but gain and feed efficiency were unaffected. The diet digestibility of major nutrients differed among dietary treatments, but how they ranked depended upon method used. The AIA method provided low variation among the means and results from extruded and muesli diets were somewhat similar to TFC estimates. However, the pelleted treatment digestibility results significantly differed between methods. The AIA method appears to be more valid as a method to determine fecal output in this study, so it leads to the conclusion that the pelleted treatment had improved digestibility over extruded. The cecal fermentation was generally affected the same by the pelleted and extruded dietary treatments compared to those in rabbits fed muesli diet. This suggests that the basal diet, more so than the process, had a bigger effect on the material arriving at the cecum for fermentation. The data also suggests that there was not a meaningful difference between pelleted and extruded diet processes.

In conclusion pelleted and extruded diets did not appear to differ from each other or affect digestibility or cecal fermentation. But, relative to the muesli diet, each resulted in a more robust cecal fermentation.

## Figures and Tables

**Table 1:** Nutritional composition (DM basis) of muesli, pelleted, and extruded diets consumed by groups of five rabbits (15 total) during the 30-day experiment.

Item	Muesli	Pelleted	Extruded
Dry Matter, %	94.2	95.2	94.6
Organic Matter, %	88.8	87.5	86.6
Gross Energy, kcal/kg	4146	3981	4002
Crude Protein, %	17.1	14.1	14.2
Acid Hydrolysis Fat, %	5.93	2.30	2.94
NFE, % (calculated)	51. 8	43.1	42.6
Starch, % (measured)	24.55	1.38	0.70
Crude Fiber, %	16.3	30.2	29.3
Acid Detergent Fiber (ADF), %	19.5	36.4	37.1
Neutral Detergent Fiber (NDF), %	31.1	56.0	55.9
Lignin, %	3.61	2.69	3.68
Ash, %	5.79	8.08	8.52
AIA, %	1.38	1.66	1.38

Muesli: Timothy Hay, Alfalfa Hay, Oats, Soybean Meal, Oat Hulls, Ground Oats, Wheat Middlings, Ground Wheat, Sunflower Seed, Wheat, Barley, Oat Groats, Shelled Peanuts, Ground Corn, Ground Flaxseed, Dehydrated Carrots, Raisins, Dried Bananas, Dried Papaya, Safflower Seed, Ground Rice, Dried Pineapple, Dehydrated Sweet Potatoes, Pumpkin Seed, Dried Cane Molasses, Dehydrated Apples, Soy Oil, Dicalcium Phosphate, Calcium Carbonate, Salt, Algae Meal, Fructooligosaccharide, DL-Methionine, Yeast Extract, Titanium Dioxide, Yucca Schidigera Extract, Vitamin A Supplement, Choline Chloride, Mixed Tocopherols, Copper Sulfate, Ferrous Sulfate, Manganous Oxide, Zinc Oxide, Riboflavin Supplement, Vitamin B12 Supplement, Vitamin E Supplement, Niacin, Menadione Sodium Bisulfite Complex, Rosemary Extract, Citric Acid, Cholecalciferol, Calcium Pantothenate, Pyridoxine Hydrochloride, Thiamine Mononitrate, Calcium Iodate, Biotin, Folic Acid, Dried A. Oryzae Fermentation Extract, Dried Bacillus Licheniformis Fermentation Product, Dried Bacillus Subtilis Fermentation Product, Cobalt Carbonate, Sodium Selenite, FD&C Yellow #5, FD&C Red #40, FD&C Blue #1, FD&C Red #3; Pelleted&Extruded: Timothy Grass Meal, Soybean Hulls, Soybean Meal, Cane Molasses, Wheat Middlings, Sodium Bentonite, Soybean Oil, Salt, Lignin Sulfonate, Hydrolyzed Yeast, Choline Chloride, Vitamin E Supplement, Zinc Sulfonate, Yeast Culture, Zinc Proteinate, Niacin, Copper Sulfate, d-Calcium Pantothenate, Vitamin A Supplement, Manganous Oxide, Riboflavin Supplement, Biotin, Thiamine Mononitrate, Magnesium Sulfate, Copper Proteinate, Sodium Selenite, Manganese Proteinate, Pyridoxine Hydrochloride, Folic Acid, Vitamin D3 Supplement, Cobalt Carbonate, Vitamin B12 Supplement, Calcium Iodate.

**Table 2:** Feed intake, weight change, and feed efficiency of rabbits fed the dietary treatments during the 30-day collection period.

Item	Muesli	Pelleted	Extruded	SEM	*p*-Value
Feed intake (DM, g/day)	91.9 ^b^	125.6 ^a^	130.4 ^a^	3.4492	<0.0001
Weight change (g)	84.4	−40.8	−92.5	124.81	0.6012
Feed efficiency (g weight change/g feed intake)	0.031	−0.007	−0.020	0.0324	0.5232

^a,b^ Means in a row with unlike superscripts differ (*p* < 0.05).

**Table 3:** Fecal output on dry matter basis of rabbits fed muesli, pelleted, or extruded dietary treatments.

Item	Muesli	Pelleted	Extruded	SEM	*p*-Value
Dry Matter, g/d	26.7 ^c^	58.6 ^a^	43.6 ^b^	1.218	<0.0001
Organic Matter	22.4 ^c^	49.6 ^a^	36.8 ^b^	1.0056	<0.0001
Energy, kcal/day	106.7 ^c^	225.5 ^a^	171.4 ^b^	5.002	<0.0001
Crude Protein, g/d	3.25 ^c^	6.51 ^a^	4.71 ^b^	0.216	<0.0001
Crude Fat, g/d	0.200 ^c^	0.488 ^a^	0.352 ^b^	0.0325	0.0002
NFE, g/d	10.5 ^c^	21.6 ^a^	16.1 ^b^	0.43	<0.0001
Starch, g/d	0.0716	0.0471	0.0349	0.01927	0.4182
Crude Fiber, g/d	9.39 ^c^	22.66 ^a^	17.00 ^b^	0.5124	<0.0001
ADF, g/d	12.3 ^c^	30.5 ^a^	22.8 ^b^	0.6461	<0.0001
NDF, g/d	18.3 ^c^	37.3 ^a^	29.1 ^b^	1.036	<0.0001
Lignin, g/d	2.90 ^b^	4.89 ^a^	3.51 ^b^	0.3607	0.0062
Ash, g/d	2.60 ^c^	6.00 ^a^	4.33 ^b^	0.1761	<0.0001
AIA, g/d	1.36 ^b^	2.80 ^a^	1.57 ^b^	0.058	<0.0001

^a,b,c^ Means in a row with unlike superscripts differ (*p* < 0.05).

**Table 4:** Apparent total tract digestibility of muesli, pelleted, or extruded dietary treatments fed to rabbits and determined by total fecal collection.

Item(%)	Muesli	Pelleted	Extruded	SEM	*p*-Value
Dry Matter	70.9 ^a^	53.2 ^b^	66.3 ^a^	1.61	<0.0001
Organic Matter	72.4 ^a^	54.4 ^b^	67.0 ^a^	1.58	<0.0001
Energy	72.0 ^a^	54.7 ^b^	66.9 ^a^	1.58	<0.0001
Crude Protein	79.3 ^a^	63.0 ^b^	74.5 ^a^	1.60	<0.0001
Crude Fat	96.3 ^a^	82.9 ^c^	90.7 ^b^	1.29	<0.0001
NFE	47.4 ^b^	53.8 ^a^	55.2 ^a^	1.50	0.0068
Starch	99.7	97.2	96.3	1.12	0.1231
Crude Fiber	37.2 ^b^	40.0 ^b^	55.2 ^a^	2.27	0.0002
ADF	31.2 ^b^	33.0 ^b^	52.7 ^a^	2.50	<0.0001
NDF	36.0 ^c^	46.6 ^b^	59.8 ^a^	2.41	<0.0001
Lignin	12.5 ^a^	−46.2 ^b^	26.7 ^a^	11.619	0.0019
Ash	51.2 ^b^	40.6 ^c^	60.8 ^a^	2.4374	0.0003

^a,b,c^ Means in a row with unlike superscripts differ (*p* < 0.05).

**Table 5:** Digestibility of muesli, pelleted, or extruded dietary treatments fed to rabbits and determined by acid insoluble ash as an internal marker to calculate apparent total tract digestibility [7].

Item (%)	Muesli	Pelleted	Extruded	SEM	*p*-Value
Dry Matter	67.7 ^b^	71.5 ^a^	62.0 ^c^	0.44	<0.0001
Organic Matter	69.2 ^b^	72.2 ^a^	62.6 ^c^	0.44	<0.0001
Energy	68.5 ^b^	72.2 ^a^	62.3 ^c^	0.40	<0.0001
Crude Protein	76.8 ^a^	77.3 ^a^	70.8 ^b^	0.78	<0.0001
Crude Fat	95.9 ^a^	89.6 ^b^	89.4 ^b^	0.73	<0.0001
NFE	76.5 ^a^	76.2 ^a^	67.9 ^b^	0.50	<0.0001
Starch	99.6	98.3	95.3	1.17	0.0623
Crude Fiber	29.2 ^c^	63.2 ^a^	48.9 ^b^	1.21	<0.0001
ADF	22.6 ^c^	58.8 ^a^	45.9 ^b^	1.01	<0.0001
NDF	27.9 ^c^	67.3 ^a^	54.0 ^b^	1.87	<0.0001
Lignin	1.68	11.28	15.64	5.820	0.2608
Ash	45.1 ^c^	63.6 ^a^	55.2 ^b^	0.93	<0.0001

^a,b,c^ Means in a row with unlike superscripts differ (*p* < 0.05).

**Table 6:** Cecal pH, ammonia, and total and individual short chain fatty acids (expressed as molar proportions) from rabbits fed muesli, pelleted or extruded dietary treatments.

Item	Muesli	Pelleted	Extruded	SEM	*p*-Value
pH	7.02 ^a^	6.38 ^b^	6.42 ^b^	0.083	0.0002
NH3, mM	3.6 ^b^	4.9 ^a^	4.3 ^a,b^	0.259	0.0142
SCFA Total, mM	11.7 ^b^	19.0 ^a^	18.5 ^a^	1.33	0.0033
Acetate, %	77.3	76.9	79.3	0.91	0.1857
Propionate, %	10.00 ^a^	7.53 ^b^	6.60 ^b^	0.54	0.0017
Butyrate, %	8.4 ^b^	12.4 ^a^	11.4 ^a,b^	0.993	0.0373
Isobutyrate, %	1.03 ^a^	0.43 ^b^	0.59 ^b^	0.085	0.0039
Isovalerate, %	1.91	1.68	1.46	0.207	0.3406
Valerate, %	1.03 ^a^	0.68 ^b^	0.65 ^b^	0.066	0.0017

^a,b^ Means in a row with unlike superscripts differ (*p* < 0.05).
